# Abortion occurs during double fertilization and ovule development in *Paeonia ludlowii*

**DOI:** 10.1007/s10265-021-01366-5

**Published:** 2022-01-20

**Authors:** Tingqiao Chen, Mengyu Xie, Yumeng Jiang, Tao Yuan

**Affiliations:** School of Landscape Architecture, National Engineering Research Center for Floriculture, Beijing Laboratory of Urban and Rural Ecological Environment, Key Laboratory of Genetics and Breeding in Forest Trees and Ornamental Plants of Ministry of Education, Beijing Key Laboratory of Ornamental Plants Germplasm Innovation and Molecular Breeding, Beijing Forestry University, Beijing, 100083 People’s Republic of China

**Keywords:** Double fertilization, Endosperm abortion, Ovule, Seed abortion, *Paeonia ludlowii*, Tree peony

## Abstract

*Paeonia ludlowii* (Stern & Taylor) D.Y.Hong, an endangered species, is indigenous to Tibet, China and propagated only by seed under natural conditions. Its natural reproduction is constrained by low fecundity. Excess seed abortion is a key factor restricting its natural reproduction, cultivation, introduction, and protection. Understanding the specific origin and occurrence of aborted ovules is important for the protection of offspring. Using serial sectioning analysis, we studied the process of pollination and fertilization of *P. ludlowii* and examined the characteristics of aborted ovules, developmental differences after flowering of normal and aborted ovules, and their ratios at different positions in *P. ludlowii* ovaries. During pollination, fertilization, and seed development, ovule abortion was frequent, with a random abortion position. There were three types of abortion, namely, abnormal pistil, sterile ovules, and embryo and endosperm abortions. Of these, embryo and endosperm abortions could be divided into early abortion and middle abortion. The early aborted ovules stopped growing on day 12, the endoblast and endosperm in the embryo sac aborted gradually. Furthermore, the shape of the embryo sac cavity changed. The volume of aborted ovules was significantly different from that of fertile ovules. At ripening, the external morphology of different types of aborted seeds was significantly different. The possible reasons for the abortion of the ovules are also discussed.

## Introduction

**Fig. 1:**
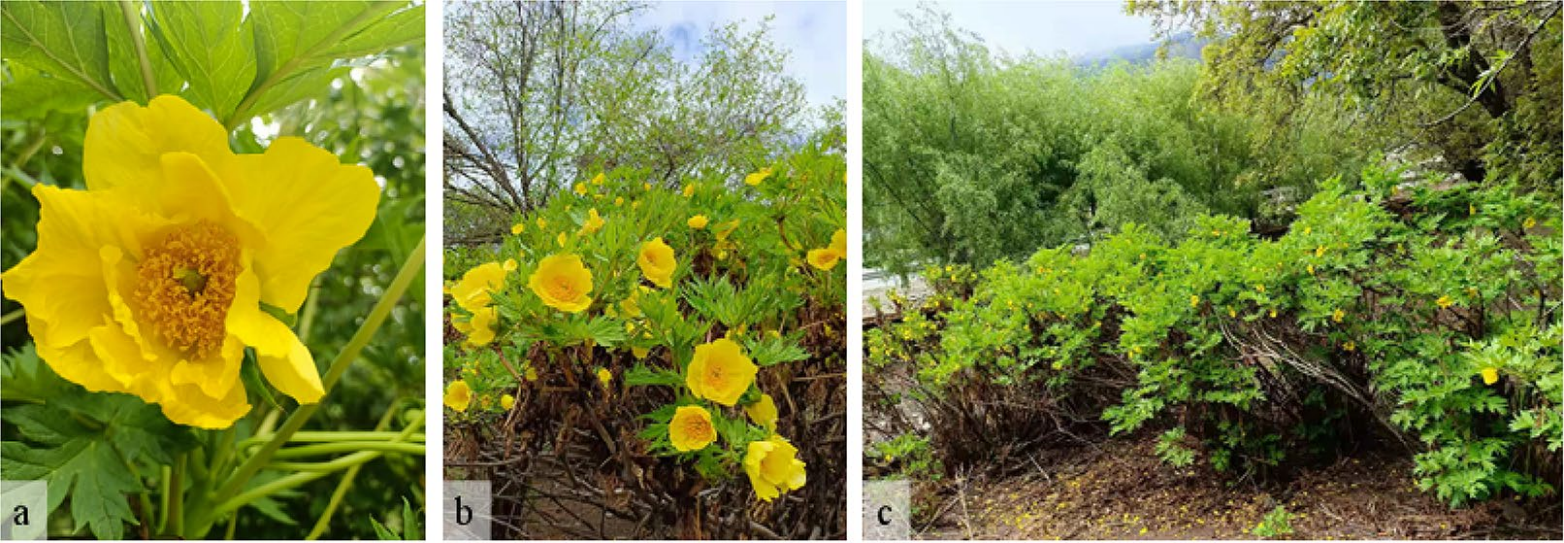
*Paeonia ludlowii*: photo taken in Nyingchi, Tibet on May 10, 2021

*Paeonia ludlowii* (Stern & Taylor) D.Y.Hong, *Paeonia* Sect. *Moutan* DC., has high ornamental, economic, breeding, medicinal, and development values (Fig. 1) (Hong 1997; Li et al. 2018; Li and Wang 2019; Lu et al. 2020, 2021; Zhang et al. 2020). Until the end of the twentieth century and the beginning of the twenty-first century, *P. ludlowii* was listed as an independent species (Hong 1997; Hong and Pan 2005). Wild *P. ludlowii* was only distributed in a small area in the eastern Himalayas, Tibet, China; to date, only six wild populations have been reported (Hong et al. 2017). All known *P. ludlowii* species originate from these regions (Cheng et al.^1998^).This species has been introduced into several areas in China, but it does not flourish in all areas; in fact, in only four areas, the species flowers and bears fruit normally (Cui et al. 2019; Li 2005; Ni 2009).

Under natural conditions, *P. ludlowii* propagates through seeds, hence, it needs to produce a large number of seeds and germinate easily to expand its population. Although *P. ludlowii* is capable of selfing and outcrossing, its rate of seed setting is only approximately 29.01%, germination percentage is 2.3%, and it requires approximately 2–3 years to grow into seedlings (Hao et al. 2014; He 2008; Tang et al. 2021; Yang et al. 2007). Moreover, due to over-excavation, catastrophes, and severe anthropogenic environmental destruction, wild *P. ludlowii* is endangered in China and is listed as a second level species in the newly released list of China’s national key protected wild plants (National Forestry and Grassland Administration of China 2021). Therefore, it is necessary to study seed abortion in *P. ludlowii*.

Seed abortion is common in most flowering plants and is considered a potentially beneficial mechanism for improving the quality of offsprings (Arathi et al. 1999; Burd 1998; Li et al. 2021; Meyer et al. 2014; Miyajima et al. 2003; Oneal et al. 2016; Satoki and Tomomi 2009). However, the mechanism of abortion severely restricts the proliferation, breeding, introduction, and conservation of endangered species. In fact, the phenomenon of ovule abortion in tree peonies (*P. suffruticosa*) is common. The abortion rate of wild *P. jishanensis* could be as high as 85% (Luo et al. 1998), and that of *P. rockii* is 50% (Cheng 1996). Existing research indicates that the amount of pollen with normal development is substantial and that it could meet the demand of all ovules in each carpel. Moreover, most of the tree peony ovules develop normally (Cheng and Aoki 1999; Pan et al. 1999), but the surface structure of the stigma is primitive and simple, which is not conducive to pollination (Zhao 2002). Hence, it has been suggested that abnormal pollination, fertilization, and seed development might lead to ovule abortion in tree peonies.

A seed is the result of ovule development, and hence, to investigate seed abortion, ovule development must first be fully understood. Therefore, in this study, fluorescence microscopy and scanning electron microscopy (SEM) were used to observe the processes of pollen tube growth and fertilization to elucidate sexual reproduction in *P. ludlowii* after pollination. We also selected paraffin sections to explore the internal structure of the embryo sac before and after ovule fertilization to determine the differences between fertile and aborted ovules and the specific origin and timing of aborted ovules. We compared fertile and abortive ovules by examining the occurrence and developmental characteristics of abortive ovules and their number and positions in a mature ovary. This study provides primary data regarding the proliferation and breeding of *P. ludlowii* and lays a theoretical foundation for further studies on seed abortion.

## Materials and methods

The materials used in this study were collected from the ex-situ Conservation Center of Chinese Paeoniaceae Wild Species (Little Red Village, Sanchuan Town, Luanchuan County, Luoyang City, Henan Province, China) (111° 21′ 36″ E, 33° 56′ 05″ N; 1,245 m altitude). *Paeonia ludlowii* seeds were sown in the autumn of 2002 (the seeds had been collected from a wild population). At the start of this study, the plants had been growing vigorously, and flowering and fruiting was stable, with nearly 1,500 flowers blooming every year. Light management was applied, and weeding was done once a year.

The experiment was conducted from May to September 2019 and from May to September 2020. The experiment was repeated twice for 2 consecutive years.

### Analysis of paraffin sections

The carpels of *P. ludlowii* were collected before the flowers had bloomed. After collection, the stigma, style, and ovary were cut off. The styles and ovaries were placed in Carnoy’s solution [95% ethanol:glacial acetic acid (v/v), 3:1]. After 12 h of vacuum treatment, the materials were transferred to 70% ethanol solution and stored in a refrigerator at 4 °C. The stigmas were then placed in a fixative [50% ethanol:acetic acid:formaldehyde (v/v/v) = 90:5:5]. After 48 h of vacuum treatment, the materials were washed twice in 50% ethanol solution, each time for 2 h, and then transferred to 70% ethanol and stored in a refrigerator at 4 °C. After proper trimming, the fixed style and ovary samples were embedded in conventional paraffin sections, and the samples were sectioned using a microtome (RM2235; Leica, Germany) to a thickness of 8 μm. The sections were stained with hematoxylin, and permanent preparations were generated using Canada balsam. Images were captured under a microscope (CX40-RFL; SDPTOP, Ningbo, China).

### Analysis of SEM

The fixed stigma samples were transferred to ethanol solutions of different concentrations (70%, 80%, 90%, 95%, and 100%) for gradient dehydration and soaked for 15 min at each concentration. The samples were then transferred to tertiary butyl alcohol, and subjected to cryodesiccation. The samples were then placed in an ion sputter coater and gilded for 20 min. The stigmas were observed by SEM (S-3400N; Hitachi, Japan).

### Pollen tube growth

The carpels of *P. ludlowii* were collected at 1 h, 3 h, 5 h, 8 h, 12 h, 24 h, 36 h, 48 h, 60 h, 72 h, 84 h, 96 h, 108 h, and 120 h after the flowers had bloomed. The carpel samples were fixed in Carnoy’s solution [95% ethanol:glacial acetic acid (v/v), 3:1] for 12 h under vacuum treatment, and then stored in 70% alcohol at 4 °C in a refrigerator.

The growth of the pollen tube through the stigma and style was observed using the aniline blue compression method (Cheng et al. 2015; Kho and Baër 1968). The collected carpel samples were dissected in the middle along the dorsal and abdominal sutures and divided into two parts. The carpel samples were then washed in distilled water and transferred into 8 mg L^–1^ NaOH solution for 2 h. Thereafter, the samples were rinsed in buffer solution of pH 6.7 (1 mol L^–1^ NaOH solution mixed with 45% glacial acetic acid; pH adjusted to 6.7) for 20 min, and then dyed with 0.1% water-soluble aniline blue dye for 6–10 h. The carpel samples were carefully taken out and spread on the slide. A drop of aniline blue solution was added, and the sample was covered with a cover glass and pressed gently. The growth of the pollen tube in the stigma and style were observed, and photographs were taken using a fluorescence microscope (DM2500; Leica).

The growth of the pollen tube in the style, ovary, and ovule was observed by section fluorescence, referring to the method of Gao et al. (2015b), with slight modifications. The specific methods were as follows. According to the pollen tube fluorescence observations in the early stage, the carpel samples after the pollen had reached the style tract were selected and then trimmed and embedded in conventional paraffin sections. After proper trimming, the fixed style and ovary samples were embedded according to the conventional paraffin section method mentioned above. The samples were sectioned using a microtome (RM2235; Leica) to a thickness of 14 μm. After dewaxing, the carpels were soaked in pH 6.7 buffer for 20 min, stained with 0.1% aniline blue solution for 2 h, and then observed and photographed under a fluorescence microscope (DM2500; Leica).

### Observation of double fertilization and seed development

The carpels at 48 h, 60 h, 72 h, 84 h, 96 h, 108 h, 120 h, 148 h, 168 h, 8 days, 9 days, 10 days, 11 days, 12 days, 13 days, 15 days, 20 days, 25 days, 30 days, 35 days, 40 days, 45 days, 50 days, 55 days, 60 days, 65 days, 70 days, 75 days, 80 days, 90 days, and 100 days after flower blooming were collected. After proper trimming, the carpels were fixed, embedded, and sectioned, following the conventional paraffin sectioning method mentioned above (slice thickness: 9–12 μm). The sections were stained with hematoxylin or saffron-solid green, and permanent preparations were generated using Canada balsam. Images were captured under a microscope (CX40-RFL; SDPTOP).

### Abortion location and abortion rate statistics

When the seeds of *P. ludlowii* were mature, 60 fruits were randomly selected to determine the number of normal mature seeds, number of aborted seeds, and abortion location in the ovary. The aborted seeds were located in the upper, middle, and lower parts of the ovary. The lower part of the ovary was connected to the fruits, the upper part was near the stigma. The abortion rate was then calculated using Eq. 1:

1 $$Abortion\;rate = \frac{number\;of\;aborted\;seeds}{{number\;of\;mature\;seeds + number\;of\;aborted\;seeds}} \times 100$$

Note: abortion seeds were undeveloped, flat, empty, brown, and had aborted embryos.

### Statistical analysis

The rate of ovule abortion was analyzed using one-way analysis of variance with IBM SPSS Statistics (version 19.0; IBM Corp., Armonk, NY). A *P* value of < 0.05 indicated significant difference. There was extremely significant difference at *P* value of < 0.01.

## Results

### Normal ovule fertilization and seed development

#### Morphological characteristics of the carpel and embryo sac:

**Fig. 2:**
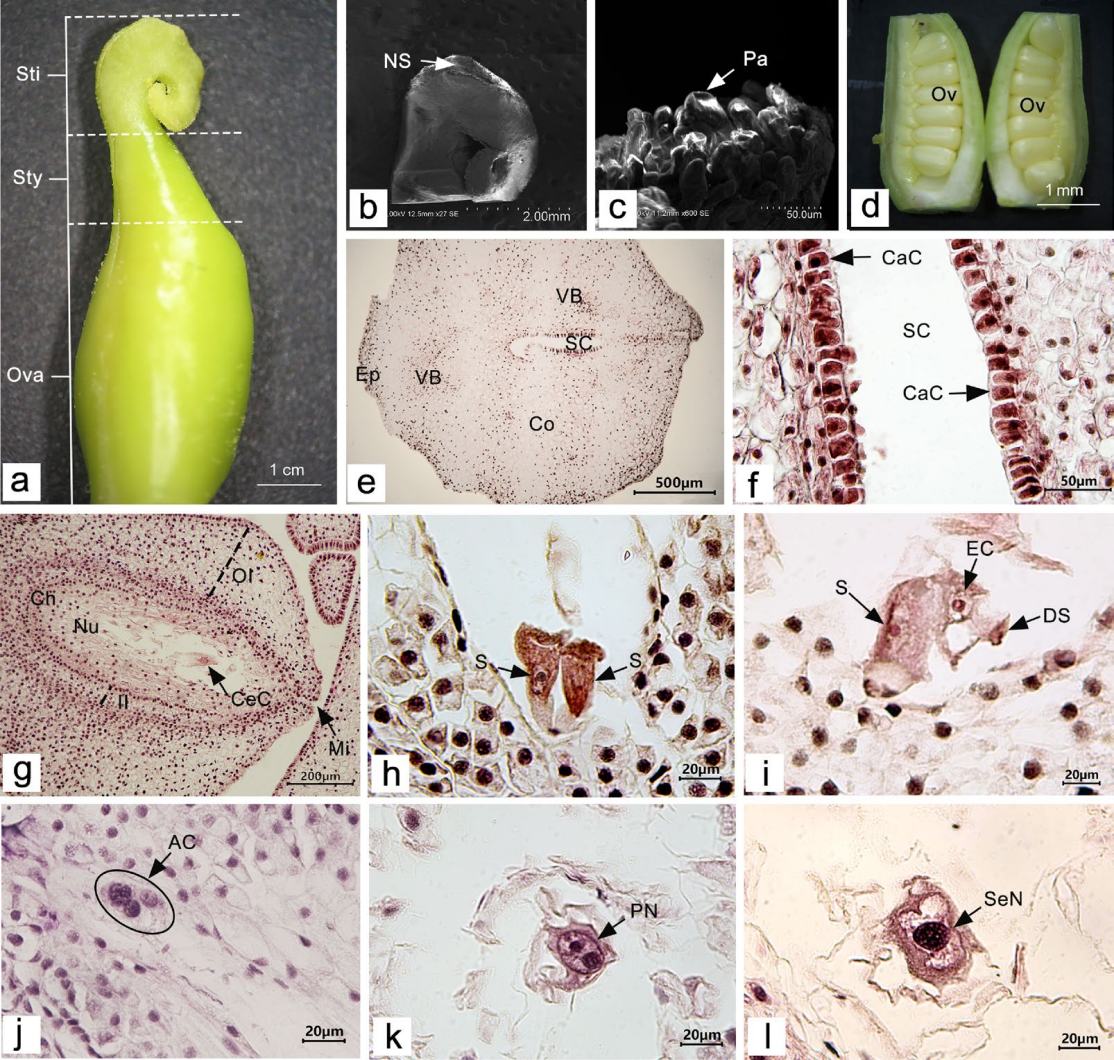
Carpel and embryo sac of *Paeonia ludlowii*: **a** the carpellate form of *P. ludlowii*, consisting of the stigma, style, and ovary; **b** stigma curling nearly 360° formed by two parts of similar size and shape, and creating a narrow strip in the middle (SEM); **c** the papillary cells on the surface of the stigma (SEM); **d** two rows of ovules in the ovary; **e** transverse section of the style, a hollow stylistic tract can be seen (Hematoxylin); **f** longitudinal section of the style, the large and distinct inner epidermal cells of the style can be clearly seen (Hematoxylin); **g** longitudinal section of an ovule showing the internal structure of the ovule (Hematoxylin); **h** the two synergids were located at the micropyle end, the nuclei were obvious, and there were obvious filamentous organelles at the micropyle end (Hematoxylin); **i** after flowering for 12 h, in the egg, one synergid had degenerated (Hematoxylin); **j** the material inside the antipodal cells is intensively stained (Hematoxylin); **k** a central cell with two polar nuclei (Hematoxylin); and **l** the central cell in which the two polar nuclei fuse to form the secondary nucleus (Hematoxylin). AC, antipodal cell; CeC, central cell; CaC, canal cell; Ch, chalaza; Co, cortex; DS, degenerated synergid; EC, egg cell; Ep, epidermis; II, inner integument; Mi, micropyle; Nu, nucellus; NS, narrow strip; OI, outer integument; Ov, ovule; Ova, ovary; Pa, papilla; PN, polar nucleus; S, synergid; Sti, stigma; Sty, style; SC, style canal; VB, vascular bundle; SeN, secondary nucleus

The carpel of *P. ludlowii* consists of three parts: stigma, style, and ovary (Fig. 2a). The plant has a wet stigma which is curled in various forms, mostly about 360°. The stigma is formed by combining two parts of similar size and shape, and a narrow band 0.1–0.4 mm in width is formed in the middle (Fig. 2b). The surface of the stigma is densely covered with papillary cells (Fig. 2c). The style is joined to the stigma, and is approximately 1–3 mm long, with a hollow style canal in the center (Fig. 2e). The style canal is the growth channel for the pollen tube of *P. ludlowii*. The nuclei of inner canal cells of the style are large and can be clearly observed. Morphologically, these cells are regular and darker than other cells. Similar to the inner tube cells of *Lilium regale* (Hu et al. 1982) and *Camellia oleifera* (Gao et al. 2019), the inner tube cells of the style canal of *P. ludlowii* are glandular (Fig. 2f). Attached to the style is the ovary, in which there is a linear arrangement of two rows of inverted ovules (Fig. 2d), with a double integument, pellicle, and thick nucellus (Fig. 2g). At blooming, the embryo sac is mature and forms a typical seven-cell and eight-nucleus structure [Fig. 2h–k; Fig. 2h, two synergids; Fig. 2i, an egg cell; Fig. 2j, three antipodal cells; Fig. 2k, central cells (two polar nuclei)]. Subsequently, the two polar nuclei fuse to form a secondary nucleus (Fig. 2l), but occasionally do not fuse to form a secondary nucleus. Only one embryo sac was observed in each ovule in all experimental materials.

#### Pollen tube growth in the style and ovary

**Fig. 3:**
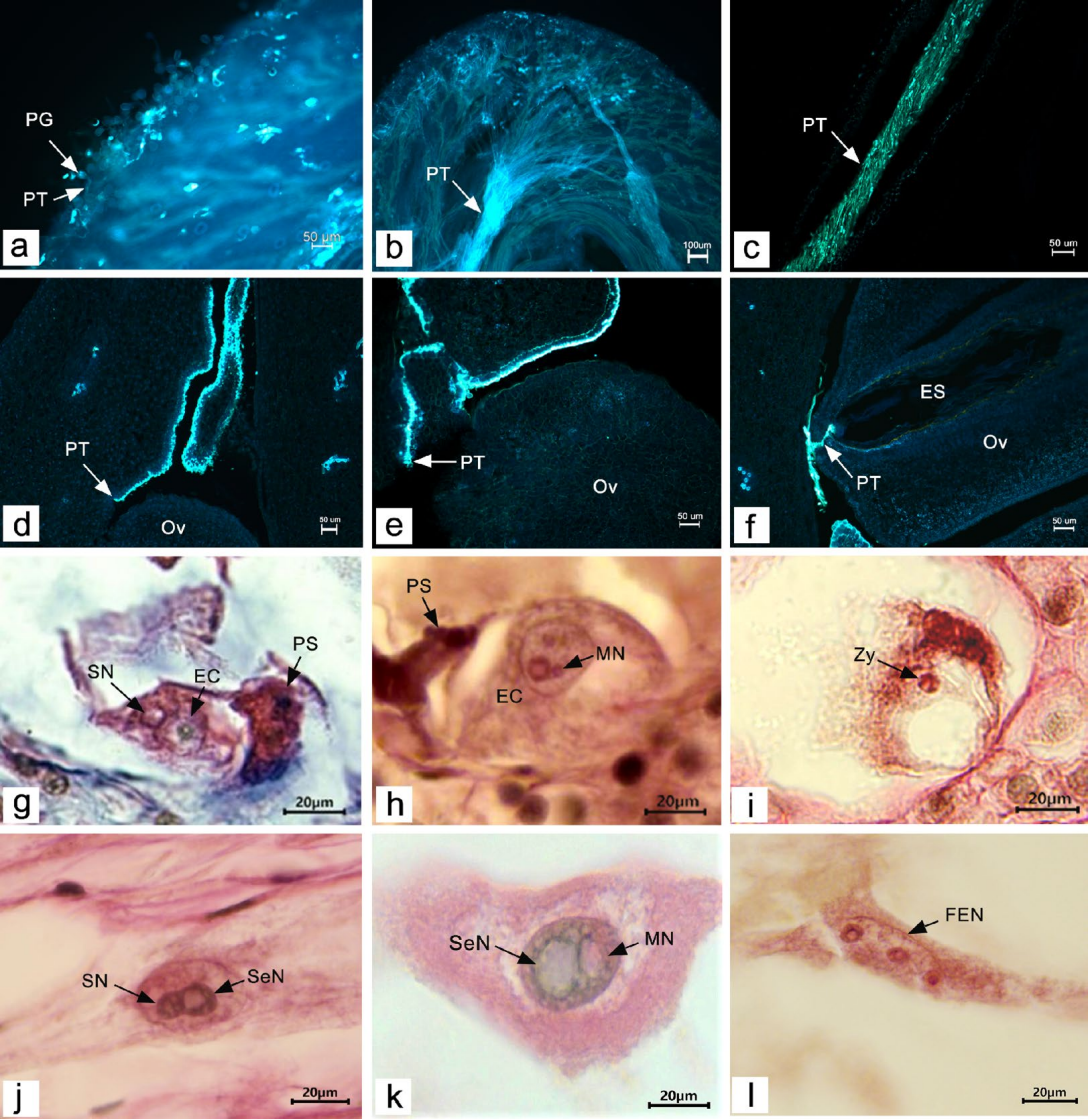
Pollen tube growth and double fertilization process after natural pollination in *Paeonia ludlowii:* **a** pollen tubes germinated on the stigma and grew into the stigma (laminated fluorescence) (Aniline blue); **b** pollen tubes converged into the style and grew downward (laminated fluorescence) (Aniline blue); **c** pollen tubes grew along the stylistic tract to the ovary (section fluorescence) (Aniline blue); **d** the pollen tube entered the ovary along the style channel (section fluorescence) (Aniline blue); **(e)** pollen tube reached near the funicle of ovule along the placenta (section fluorescence) (Aniline blue); **f** pollen tube entered the embryo sac through the nucellus (section fluorescence) (Aniline blue); **g** the sperm nucleus attached to the egg nucleus (Hematoxylin); **h** the sperm nucleus entered the egg nucleus, showing the male nucleoli (MN) in the egg nucleus (Hematoxylin); **i** zygote formation (Hematoxylin); **j** the spermatic nucleus attached to the secondary nucleus (Hematoxylin); **k** male nucleoli appeared in the polar nucleus (Hematoxylin); and **l** free endosperm nucleus formation (Hematoxylin). EC, egg cell; ES, embryo sac; FEN, free endosperm nucleus; MN, male nucleus; Ov, ovule; PG, pollen grain; PT, pollen tube; PS, persistent synergid; SN, sperm nucleus; SeN, secondary nucleus; Zy, zygote

The flowers of *P. ludlowii* open during the day and close at night, a feature that facilitates pollination by wind and insects. We observed that 1–12 h after flowering, pollen grains fell on the stigma and germinated, the pollen tube penetrated deep into the stigma, where the pollen remained (Fig. 3a). After entering the stigma, the pollen tubes grew along the vascular bundles of the stigma and converged into the style (Fig. 3b). The tube then grew to the base through the stylistic tract and the layer of mucous secreted by the inner epidermal gland cells (Fig. 3c). After 36–48 h of flowering, the pollen tube reached the base of the style (Fig. 3d) and continued to grow toward the ovary. After penetrating the ovary, the pollen tube continued to grow along the placenta’s epidermal cells (Fig. 3e). After 48–60 h of flowering, when the pollen tube approached the ovule, it was bent nearly 90° to approach and penetrate the micropyle and pass through the nucellus into the embryo sac (Fig. 3f). A few aborted pollen grains, as well as some abnormal pollen tubes and self-pollinating pollen tubes could be seen on the stigma after pollination. However, due to the large amount of pollen, sufficient pollen tubes entered the ovary, enabling us to observe each ovule in the ovary with the pollen tube entering.

#### Double fertilization

After passing through the apical nucellus from the degenerated synergid entering the embryo sac, the pollen tube released two sperm cells, one fusing with the egg nucleus and the other with the secondary nucleus (polar nucleus) of the central cell. The sperm nucleus gradually approached the egg nucleus, fused with it, and eventually formed the large nucleoli of the zygote completing the fertilization of the egg cell (Fig. 3g–i). This process was observed at 60–144 h after flowering. After zygote formation, a period of dormancy was required before the division stage. The fusion of the sperm nucleus and secondary nucleus was similar to that of the fusion of the sperm nucleus and egg nucleus (Fig. 3j, k). The difference was that the fertilization rate of the central cell was significantly higher than that of the egg cell, as determined by observing several successive sections. After fertilization of the central cell, the primary endosperm nucleus was formed; this nucleus then divided to produce the free endosperm nucleus (Fig. 3l). This process was observed from 60–108 h after flowering. The timing of double fertilization of the ovules in each ovary was not synchronous. During 108–144 h after flowering, some ovules in the double fertilization stage were also observed, but the number was small; the process was completed by day 7 after flowering.

**Fig. 4:**
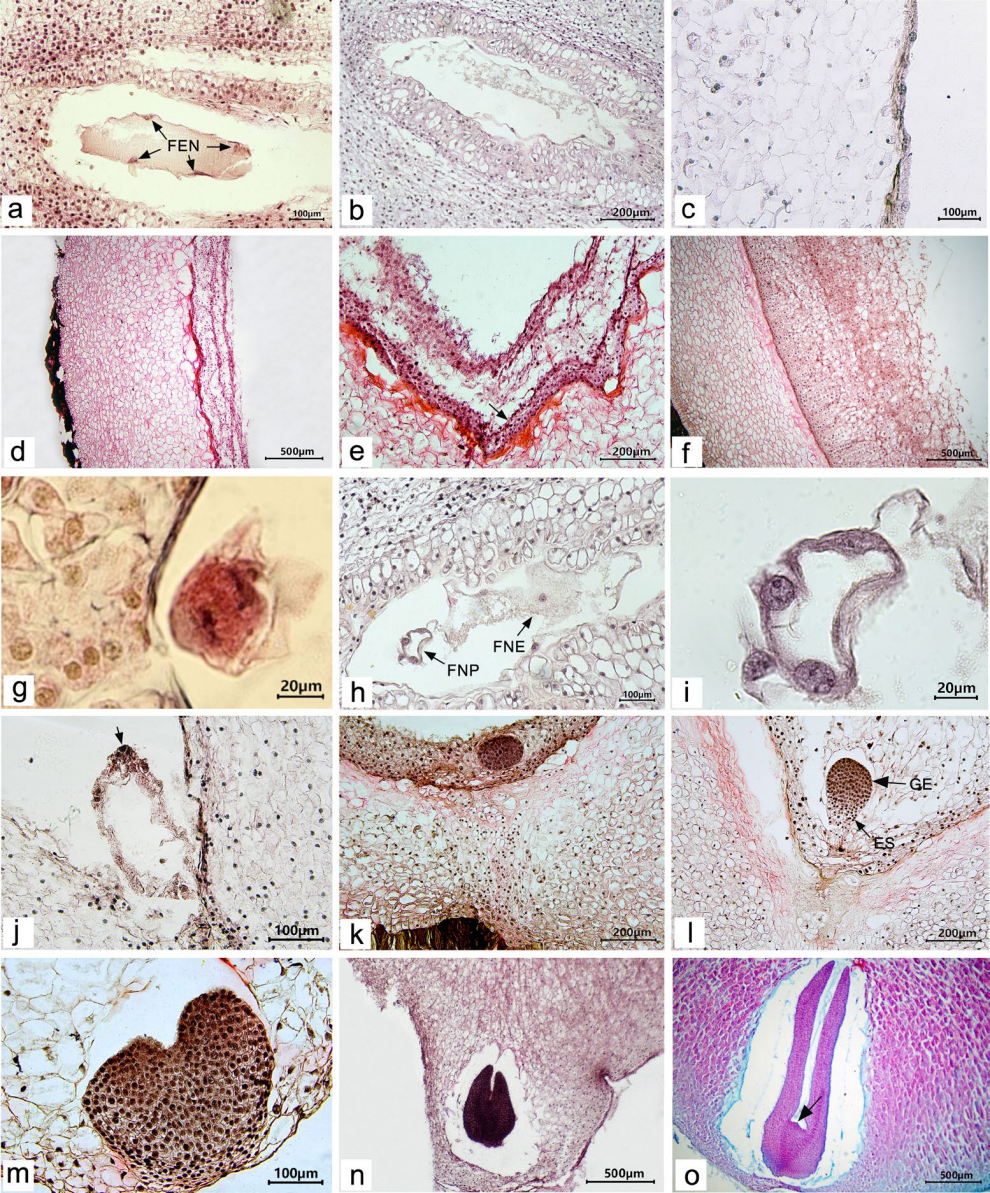
Development of the endosperm and embryo in a fertile ovule of *Paeonia ludlowii*: **a**–**c** at 120 h, 12 days, and 20 days after flowering, the free nuclei of the embryo sac and endosperm continued to divide, the nucellus and inner tegmentum degenerated, and the endosperm adjoined the outer tegmentum (Hematoxylin); **d** the free nuclei of the endosperm continued to divide, the degraded area of the pearly layer was formed (40 days after flowering) (Hematoxylin); **e** 45 days after flowering, the endosperm began to cellularize (arrow shows the cellularized endosperm) (Hematoxylin); **f** 55 days after flowering, the endosperm was cellularized (Hematoxylin); **g** telophase of the first zygote division (108 h after flowering) (Hematoxylin); **h**, **i** the embryo sac of free nuclear proembryo and free nuclear endosperm (12 days after flowering), **i** the amplification of free nuclear proembryo in **h** (arrow shows free nuclei) (Hematoxylin); **j** the free nuclei at the chalazal end began to cellularize (30 days after flowering) (arrow shows the cellularized embryo) (Hematoxylin); **k** early stage of spherical embryo (55 days after flowering) (Hematoxylin); **l** late globular embryo (60 days after flowering) (Hematoxylin); **m** heart-shaped embryo (65 days after flowering) (Hematoxylin); **n** torpedo embryo (70 days after flowering) (Hematoxylin); **o** cotyledons at seed maturity (arrow indicates points of growth) (100 days after flowering) (Hematoxylin). FNN, free nuclear endosperm; FNP, free nuclear proembryo; GE, globular embryo; ES, embryo stalk

During the late development of fertile ovules in *P. ludlowii*, the primary endosperm nucleus was the first to change. After the fertilization of the secondary nucleus (polar nucleus), the primary endosperm nucleus split to form several free endosperm nuclei inside the embryo sac (Fig. 4a). Subsequently, the free endosperm nuclei were divided repeatedly, and the number of free nuclei increased continuously, during which no cell wall was formed (Fig. 4b–d). Until 45 days after flowering, the free nuclear endosperm began to cellularize, at which point the internal ovule was liquid or semi-liquid (Fig. 4e). The cellularization was completed at approximately 55 days after flowering (Fig. 4f). Thereafter, the volume of the endosperm increased rapidly; the endosperm reached its final shape and size approximately 75 days after flowering and the interior of the ovule gradually changed from liquid or semi-liquid to a solid state.

The endozygote of the fertile ovules began to divide after the end of dormancy (Fig. 4g). The zygote divided first to form the binuclear proembryo and then divided repeatedly and synchronously. The number of free nuclei increased continuously, and the proembryo grew gradually. During this period, no cell wall was formed (Fig. 4h, i). The free nuclear stage of the proembryo lasted from zygotic dormancy to 30 days after flowering. The free nuclei then began to cellularize, generally beginning at the chalazal end and advancing toward the micropylar end (Fig. 4j). At 55 days after flowering, embryo development reached the globular embryo stages (Fig. 4k, l). Thereafter, the embryonic somatic cells divided and differentiated rapidly, and the embryonic development successively went through the heart-shaped embryo (Fig. 4m), torpedo-shaped embryo stage (Fig. 4n), and cotyledon embryo stage (Fig. 4o), until the final seeds were mature.

### Abnormalities during fertilization and seed development

#### Abnormal pistil

**Fig. 5:**
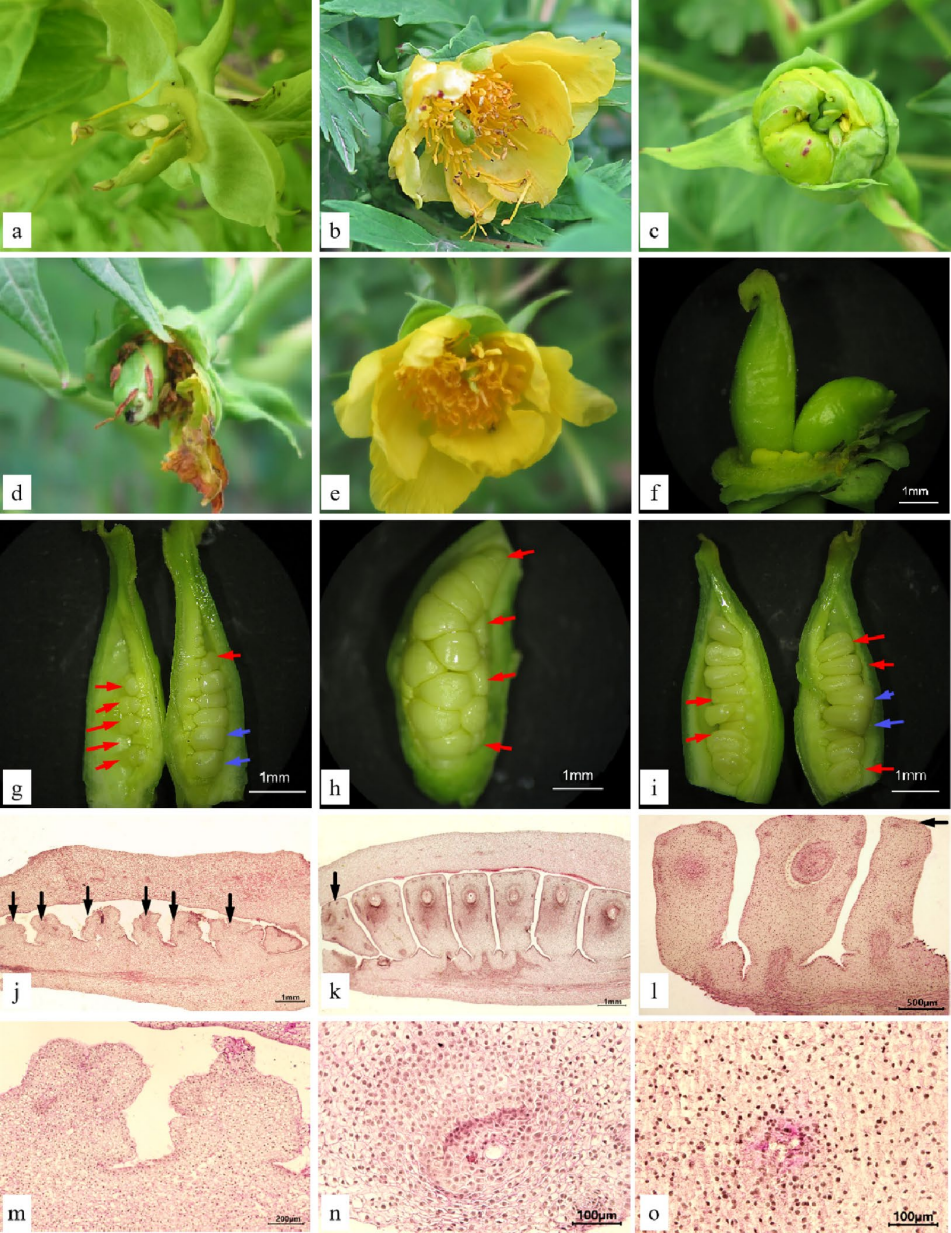
Abnormal pistils and sterile ovules of *Paeonia ludlowii*: **a** a ruptured ovary; the ovules are protruding; **b** stigma; **c** abnormally curved carpel; **d** abnormal style and stigma; **e**, **f** one carpel of flower without stigma or with a regressed stigma; **g**–**i** sterile ovules at different position in the ovary (sterile and fertile ovules are indicated by red and blue arrows, respectively); **j–o** microstructure of abortive ovules in the ovary (Hematoxylin), **j**, **m** the whole row of ovules in the ovary were aborted (on the first day of flowering), **k**, **n** ovules in a row that were aborted in the upper position of the ovary, and **l**, **o** ovule abortion in the middle position

During the growth and development of *P. ludlowii*, some pistils were abnormal in different ways, for example, ovule bursting, caused by the rupture of the carpel (Fig. 5a), stigma exposure before anthesis (Fig. 5c), and carpel deformity (Fig. 5b, d–f).

These abnormal pistils were present before flowering and could not be fertilized normally. Among nearly 500 flowering branches and about 1,500 open flowers (some buds failed to open normally), approximately 30 had abnormal pistils, accounting for 0.02%.

#### Sterile ovule

During the study, repeated experiments and many dissections showed that the appearance of carpels in the ovaries of some test materials was the same as that of other carpels, but there were sterile ovules inside. These ovules were present on the day of flowering, when the embryo sac matured, rather than being abnormal during post-pollination development. These ovules were markedly different from other ovules in morphology; that is, they were smaller in size or deformed (Fig. 5g–i). Some had no embryo sac, and there were no clear spaces between the outer and inner integument (Fig. 5j, m). Moreover, some of the ovules had embryo sac structures, and the space was small, without any obvious organizational structure (Fig. 5k, l, n, o). In the materials observed, not all carpels had sterile ovules, and according to the statistics, the carpels with sterile ovules accounted for 10.59–15.73% of all the test materials.

In addition, through several sections and repeated experiments, we found some abnormal embryo sacs in the experimental materials during fertilization. The main characteristics of these abnormal embryo sacs were as follows. (1) At 84 h after anthesis, the pollen tube had reached the ovary, and most ovules had begun fertilization, but the two synergistic cells in the individual embryo sac remained intact without degeneration. The embryo sac, which could not enter the physiological state of fertilization in time, could not be fertilized normally (Cheng 1996). (2) At 8–12 days after flowering, free nuclear proembryo and endosperm were formed in the fertilized ovules, and the number of free nuclei increased significantly, but the secondary nuclei in some of the embryo sacs remained unfertilized, and no endosperm free nuclei were formed. These ovules could not develop into seeds normally without double fertilization. The ovules containing abnormal embryo sacs accounted for approximately 1.2% of all experimental materials, and only a few carpels had abnormal embryo sacs.

#### Degeneration of the embryo and endosperm

**Fig. 6:**
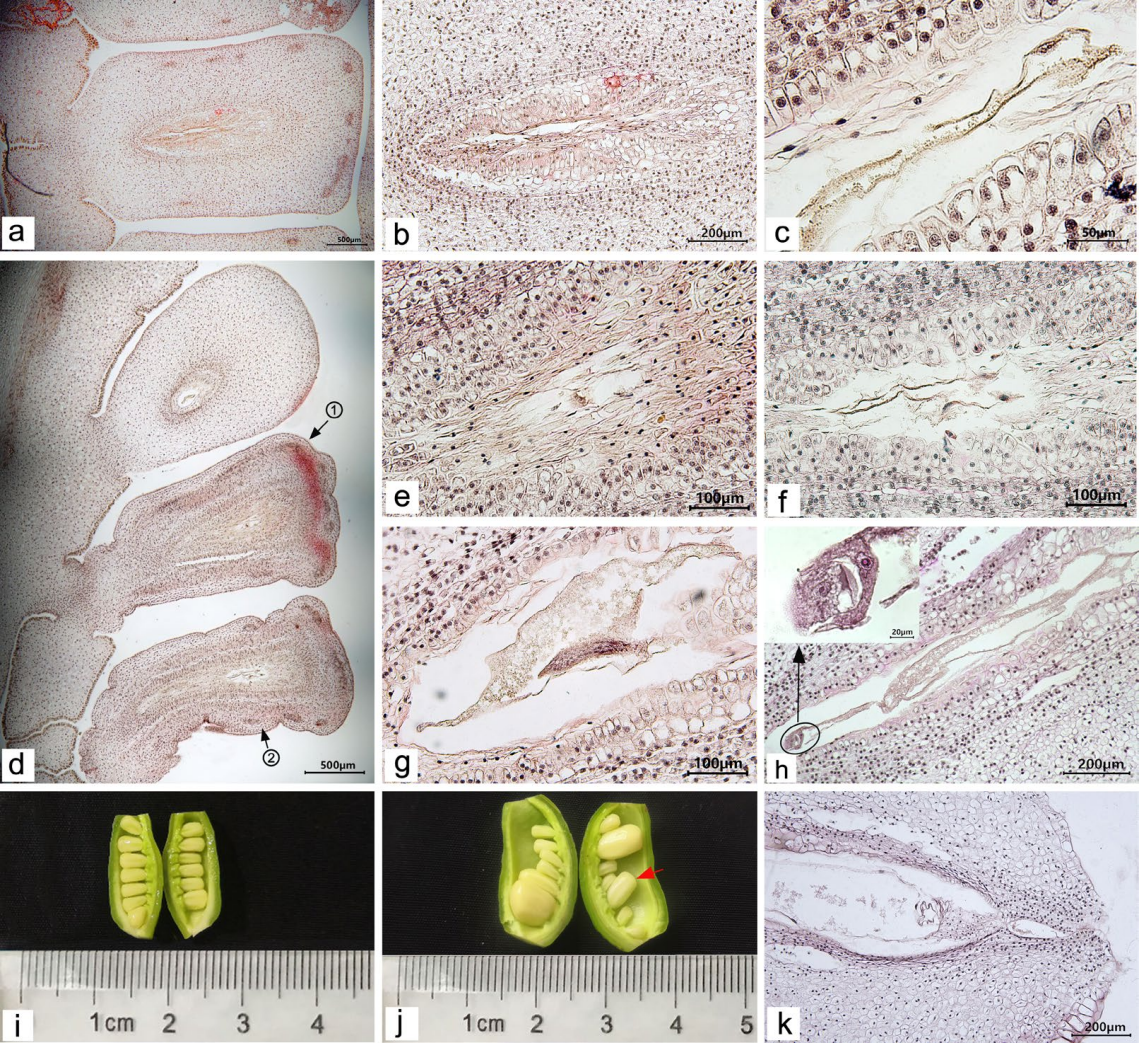
Degradation of embryo and endosperm after double fertilization of aborted ovules in *Paeonia ludlowii:* **a**, **b** the endosperm in the embryo sac was degenerative. The cavity of the embryo sac became narrow 9 days after flowering (Hematoxylin); **c** aborted ovules at 10 days after flowering (Hematoxylin); **d–f** two successive aborted ovules (10 days after flowering) (Hematoxylin), **e** enlargement of the embryo sac in d-①, **f** enlargement of the embryo sac in d-②, the cytoplasm of the endosperm was reduced, and most of the free nuclei had degenerated; **g** there was free endosperm nuclear division, but no separation (11 days after flowering) (Hematoxylin); **h** the embryo in the embryo sac was in the dikaryotic proembryo stage, but the free nucleus of the endosperm had degenerated, only a part of the cytoplasm remained, and the embryo sac became small (12 days after flowering); **i** at 12 days after flowering, the ovules volume in the ovaries was different (Hematoxylin); **j, k** at 20 days after flowering, some of normally developing ovules in the early stage were aborted, **k** the internal structure of the embryo sac shown with the red arrow in **j** (Hematoxylin)

There were some ovules in the carpel, which could complete double fertilization and showed zygote and primary endosperm nuclear division in the early stages but could not develop further in the late stages. At 9 days after flowering, some aborted ovules similar in size to normal ovules showed abnormalities in the embryo sac and degeneretion of the free nuclei of the endosperm (Fig. 6a, b). On day 10 after flowering, the free nuclei of the endosperm continued to degenerate (Fig. 6c). Some abortive ovules of the same size as normal ovules showed shrinkage and deformity in their outer tepals after dewatering and embedding (Fig. 6d). When no free nuclear embryo was observed in the embryo sac, the endosperm developed abnormally, and the free endosperm nuclei were rare and irregular. The cytoplasm of the endosperm gradually disintegrated and was in a state of severe degeneration (Fig. 6e, f). In the material at 11 days after flowering, apart from the phenomenon of endosperm abortion, the free endosperms in some of the embryo sacs aggregated after nuclear division but did not separate (Fig. 6g). At 12 days after flowering, the endosperm of the fertilized ovules degenerated. In some embryo sacs, only the free nuclear proembryo developed to the stage of dinucleus proembryo, but the free endosperm nuclei were rare, the cytoplasm was significantly reduced, and the embryo sac was in a severely degenerated state; consequently, the embryo sac became narrow (Fig. 6h). Embryo sacs were distinct from the fertile ovules at the same period (Fig. 4h). On the same day, with the development of the ovaries and the enlargement of the ovules, the ovules’ volume in the ovaries began to show differences (Fig. 6i). A few aborted ovules developed normally until approximately 20 days after flowering, and the volume of the ovules was slightly larger than that of other early aborted ovules and slightly smaller than that of fertile ovules (Fig. 6j, indicated with red arrows). The free nuclei of the embryo and endosperm in these aborted ovules were not as developed as the other fertile ovules and tended to degenerate (Fig. 6k). At this time, less than one-third of the fertile ovules were observed in the ovary, and abortive ovules were observed in all carpels. The results indicated that the abortion of embryo and endosperm occurred mainly in the early stage, 9–12 days after flowering.

#### Late development of abortive ovules

**Fig. 7:**
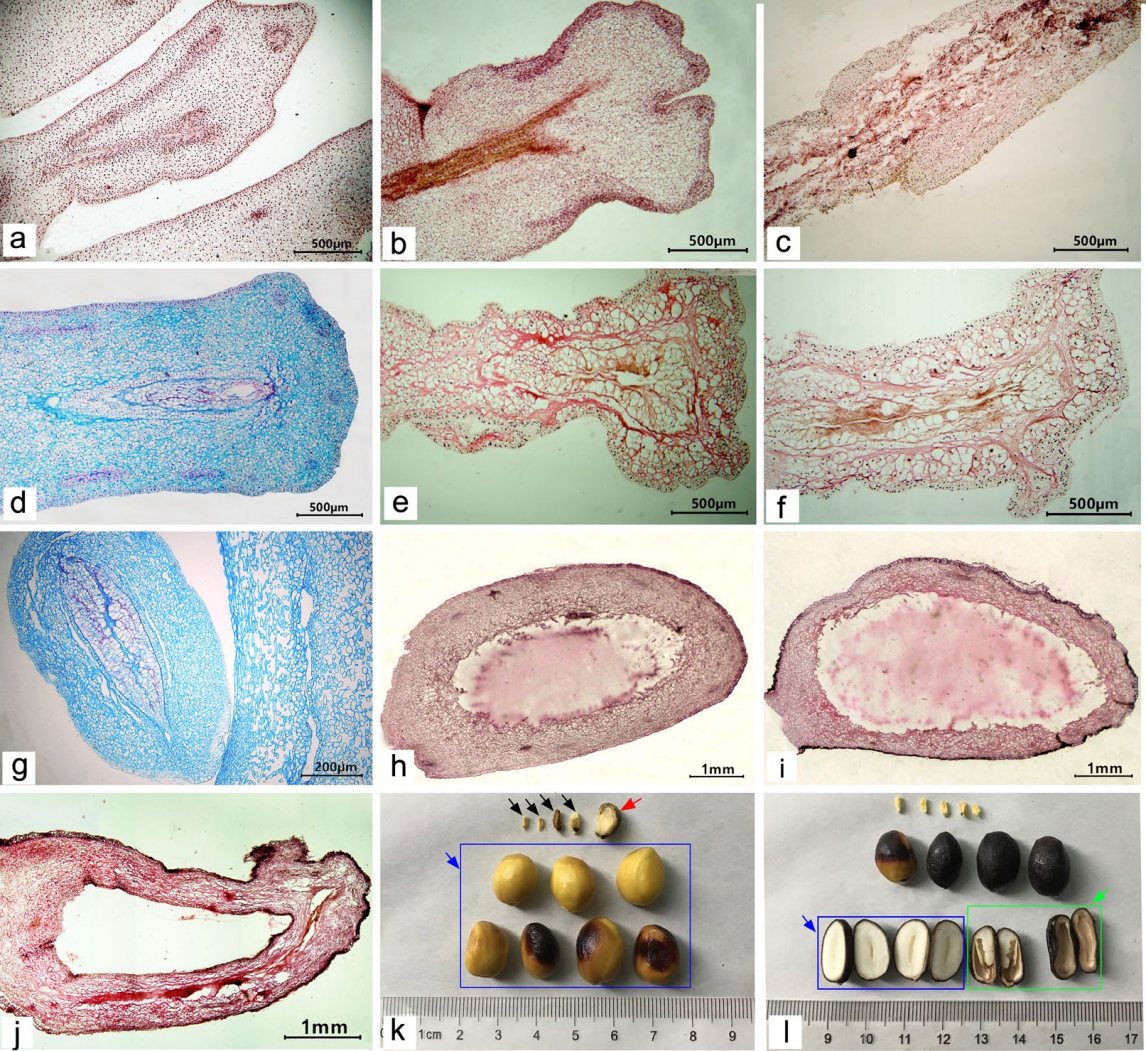
Late development of aborted ovules: **a–d** the development of aborted ovules at 12, 25, 30, and 35 days after flowering **a–c** were stained with hematoxylin and **d** was stained with saffron-solid green); **e, f** development of aborted ovules at 55 days after flowering (Hematoxylin); **g** development of aborted ovules at 70 days after flowering (saffron-solid green); **h–j** development of the aborted ovules of free nuclear embryos and endosperm aborted at 20 days after flowering at 30, 45, and 70 days after flowering, respectively (Hematoxylin); **k** comparison of fertile ovules and aborted ovules at seed harvest (normal seeds are indicated with the blue arrow, the early aborted seeds at 9–12 days after flowering are indicated with the black arrows, and the medium-term aborted ovules at 20 days after flowering are indicated with the red arrow), and **l** comparison of the internal structure of aborted ovules and fertile ovules at seed harvest (normal seeds are indicated with the blue arrow, and the late abortive seeds are indicated with the green arrow)

After 12 days, the embryo sacs of some aborted ovules gradually contracted toward the nucellus, the cavity of the embryo sac became narrow and long, degenerated, and necrotic, and filled with a brown material (Fig. 7a, b). At 30 days after flowering, the embryo, nucellar, and integument gradually degenerated (Fig. 7c). Some embryo sacs were filled with large cells, with only a narrow gap in the middle, with darker staining (Fig. 7d–g). With the growth of the ovule, the free nuclear embryo and endosperm of some ovules that began to abort at approximately 20 days after flowering degenerated completely, and the outer envelope had also gradually degenerated, finally forming a hollow shell with no embryo or endosperm in the embryo sac. The outer cover gradually degenerated, finally forming a hollow shell without an embryo and endosperm in the embryo sac (Fig. 7h–j). Although the embryo and endosperm were not formed in the aborted ovules, the external morphology of the aborted seeds significantly differed (Fig. 7k, indicated with the red arrow). In addition, when harvesting seeds, we also found a few seeds with external morphology and volume similar to those of the fertile seeds, but they were deformed after applying pressure. After peeling, the seeds were hollow inside, without embryo or endosperm structure (Fig. 7l, indicated with the green arrow). This could be attributed to embryo and endosperm atrophy during seed maturity.

### Position and rate of aborted ovules in P. ludlowii

**Fig. 8:**
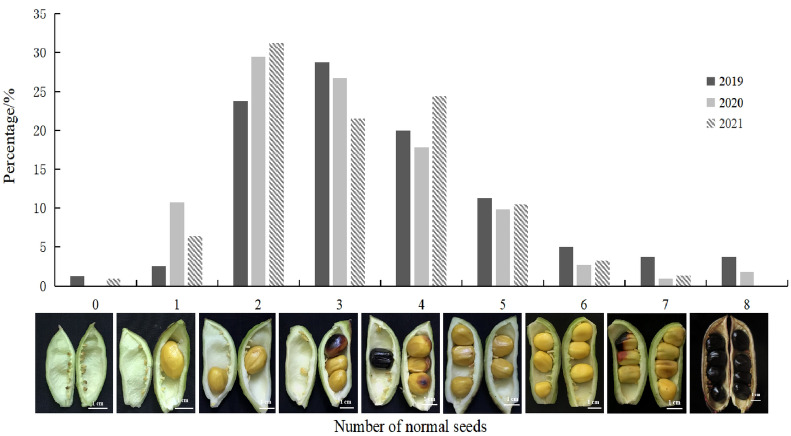
Distribution of the number of normal seeds in *Paeonia ludlowii* ovaries

The total number of seeds in a single carpel ranged from 6–23, and the number of normal seeds ranged from 0–8 (2–4 more). More than two-thirds of the ovules were aborted (Table 1, Fig. 8). According to the statistics of the rate of abortion seeds (Table 1), the seed abortion rate in the ovaries in 2019 and 2020 was 69.14% and 74.01%, respectively, and the abortion rate in wild populations was also high (66.39%), indicating that seed abortion occurred independent of the growing environment.

**Table 1:** Ovule position and abortion rate in *Paeonia ludlowii*

Year	Number of seeds in a single ovary			Position and rate (%) of ovule abortion			Mean (%)
	Total number of seeds	Normal seeds (mature seeds)	Abortion seeds	Upper part of the ovary	Middle part of the ovary	Lower part of the ovary	
2019	6–16	0–8	4–14	66.25 ± 3.20b	66.98 ± 2.66b	77.08 ± 1.65a	69.14 ± 1.41
2020	6–16	1–8	4–14	70.86 ± 2.813b	83.33 ± 2.484a	78.59 ± 1.523a	74.01 ± 1.06
2021	8–23	0–7	6–17	74.90 ± 1.93a	71.65 ± 1.76a	78.95 ± 1.64a	77.88 ± 8.52

Different lowercase letters indicate significant difference at the 0.05 level (*P* < 0.05)

The statistical results of the position of aborted seeds in *P. ludlowii* (Table 1) showed that, in 2019, the abortion rate in the lower part of the ovary was the highest (77.08%) and the abortion rates in the middle and upper parts of the ovary were 66.98% and 66.25%, respectively. Moreover, the differences among the three parts were significant. In 2020, the abortion rate was the highest in the middle part of the ovary (83.33%), followed by the lower part (78.59%) and the upper part (70.86%). These results showed that there was no specific position for seed abortion in the ovary of *P. ludlowii*, and that seed abortion was random.Moreover, in 2021, the abortion rate in the lower part of the ovary was the highest (78.95%) and the abortion rates in the upper and middle parts were 74.90% and 71.65%, respectively.

## Discussion

### *Characteristics of the carpel and embryo sac of *P. ludlowii

The carpels are plant reproductive organs, and ovary development (ovule development and embryo sac formation) and stigma and style formation are particularly important for fertilization (Gao et al. 2019; Bedinger et al. 2016). The carpels of tree peonies are composed of the ovary, style, and stigma. Zhao and Lian (2002) found that the stigma of *P. ludlowii* curled at an angle of approximately 90°. However, in this study, most of the stigmas curled into a ring at an angle of 360° or nearly 360°. The results of this study were slightly different from those of previous studies; this could be because, in order to adapt to the environment and to meet the needs of pollination, the stigma had undergone a certain degree of evolution, which enlarged the pollen surface. In addition, the electron microscopy structure of the stigma showed that the stigma surface of *P. ludlowii* is covered with a large number of papillae, which also helps the stigma to accept many pollen grains.

The style of *P. ludlowii* is hollow, with a canal in the center. As in lily and camellia, which have hollow styles, the channel is surrounded by a layer of glandular secretory cells, also known as channel cells (Gao et al. 2019; Hu et al. 1982; Zhang 2020). The style is the only way that the pollen tubes can enter the ovary. In plants with style channels, pollen tubes mainly grow along the surface of the channel cells and then enter the ovary (Gao et al. 2015a).

The ovules of *P. ludlowii* are inverted, with a double integument, thick nucellus, and polygonum-type embryo sac. The basic structure is the same as that of other peonies (Cheng and Aoki 1999; Wang et al. 2010). The embryo sac of *P. ludlowii* developed and matured earlier, with one synergistic cell degenerating at 12 h after flowering, and the embryo sac entered the fertilization state. Walters’ (1962) research on *P. californica* revealed that each ovule had 1–4 embryo sacs, and the phenomenon of multiple embryo sac development has also been found in the ovules of *P. rockii* (Cheng and Aoki 1999). However, only one embryo sac was observed in one ovule inseveral of *P. ludlowii* samples during our experiment. Wang et al. (2010) observed one embryo sac in *P. delavayi*, Further, Vinogradova and Zhinkina (2020) indicated that only one embryo sac formed in *P. veitchii* and *P. caucasica*, and they assumed that callose participates in blocking the developmental signals to neighboring megasporocytes that further arrests their development.

### Characteristics of pollen tube growth in P. ludlowii

The completion time of pollen grain germination and pollen tube growth is 2–3 h in *P. rockii* (Cheng 1996). However, the pollen grains of *P. ostii* ‘Feng Dan’ germinate immediately after falling on the stigma and form pollen tube channels 1 h after pollination, and the pollen tubes enter the ovule and reach the embryo sac within 12–48 h after pollination (Chen 2020; Dong 2010; Fan et al. 2004). In ‘High Noon’ (a distant hybrid between subgroups in the Sect. *Moutan*), germination is not observed until 8–11 days after pollination, when the pollen tube enters the nucellus (He and Cheng 2006). Here, the observation of *P. ludlowii* were similar to those of *P. ostii* ‘Feng Dan’, and the pollen tubes in *P. ludlowii* entered the embryo sac at 48–60 h after flowering. Pollen germination capacity and pollen tube growth rate in plant styles are affected by internal and external factors, such as the variety characteristics, nutritional conditions, temperature, light, and climate, as reported in some species and varieties (Dogterom et al. 2000; Lau and Stephenson 1994; Ruane 2008; Schlichting 1986; Tuell and Isaacs 2010). Thus, the germinated pollen tube takes a longer time to enter the embryo sac in *P. ludlowii* than in *P. rockii* due to varietal differences, planting conditions, and climatic conditions after pollination.

In angiosperms, the pollen tube enters the ovule in two ways, namely porogamy and chalazogamy (Li and Gao 2008). When the pollen tube of *P. ludlowii* approached the ovule, it turned nearly 90° and entered the micropyle. Therefore, the fertilization pattern of *P. ludlowii* is porogamy, as in *Chrysanthemum grandiflorum* and *Camellia oleifera* (Deng et al. 2010; Gao et al. 2015b). The double fertilization of *P. ludlowii* is consistent with that in *P. rockii* (Cheng 1996), indicating that double fertilization in *P. ludlowii* is normal. According to a report by Dong (2010), in *P. ostii* ‘Feng Dan’, two sperms enter the egg cell at the same time or near the secondary nucleus. However, we did not observe this phenomenon in *P. ludlowii*. Whether it was related to the experimental materials remains to be studied.

### Characteristics of double fertilization and development of embryo and endosperm in *P. ludlowii*

The development of endoembryo and endosperm in the fertile ovule of *P. ludlowii* is similar to that in other tree peony species (Cave et al. 1961; Cheng and Aoki 1999; Dong 2010; Mu and Wang, 1985), but the timing is significantly different (Table 2). This difference can be attributed to the plant species and environmental conditions, such as flowering and early embryo development temperatures.

**Table 2:** Initiation time of free nuclear proembryos and endosperm in *Paeonia*

Plant species	Growing environment (location)	Cellular time of proembryo (days after flowering or pollination, day)	Cellular time of endosperm (days after flowering or pollination, day)	Source
*P. californica*	Wild (California, US)	> 12–14	> 12–14	Cave et al. (1961)
*P. brownii*				
*P. lactiflora*	Cultivated (Beijing, China)	17–23	> 17–23	Mu and Wang (1985)
*P. rockii*	Cultivated (Lanzhou, China)	29	29	Cheng (1996)
*P. ostii* ‘Feng Dan’	Cultivated (Shanghai, China)	23	23	Dong (2010)
*P. ludlowii*	Cultivated (Luoyang, China)	30	45	This study

### Ovule abortion and its characteristics in *P. ludlowii*

In seed plants, abortion can be divided into nonrandom and random abortion. The former refers to a certain regularity in the position of seed abortion in the fruit, as in *Phaseolus coccineus* (Rocha and Stephenson 1990), *Robinia pseudoacacia* (Susko 2006; Yuan et al. 2014), *Caesalpinia gilliesii* (Calviño 2014), *Bauhinia ungulata* (Mena-Alí and Rocha 2004), and *Anagyris foetida* (Valtueña et al. 2010)*.* The latter means that the abortive seeds have no specific position in the fruit. Evidently, the abortion in *P. ludlowii* belongs to the latter.

The aborted ovules of *P. ludlowii* are of three types: abnormal pistils, sterile ovules, and embryo and endosperm abortions. The phenomenon of pistil abortion, such as abnormal pistils and sterile ovules, is common in nature (Hou et al. 2011; Wang 2006; Wetzstein et al. 2011). However, although pistil abortion exists, ovule abortion in *P. ludlowii* mainly involves embryo and endosperm abortions. According to the time, embryo and endosperm abortions in *P. ludlowii* could be divided into two types. (1) Early abortive, which occurred at 9–12 days after flowering and the aborted ovules stopped growing and were smaller than fertile ovules. The development of endosperm was abnormal, the free nucleus of the endosperm continued to degenerate, and the embryo sac became narrow. The number of abortive ovules in the ovary was more than 60%, and all carpels had abortive ovules. (2) Medium-term abortive, which occurred at approximately 20 days after flowering. The volume of aborted ovules was slightly larger than that of other early aborted ovules and slightly smaller than that of fertile ovules. The free nuclei of the embryo and endosperm in the embryo sac began to degenerate. Based on the proportion of aborted ovules observed in each stage, the embryo and endosperm of *P. ludlowii* were mainly aborted in the early stage, that is, 9–14 days after flowering. This occurrence time was slightly different from the results of He and Cheng (2006), which might be related to various characteristics of the plant including different pollination and fertilization times.

Several factors besides species characteristics and resource constraints affect plant embryo abortion (Brookes et al. 2008; Florez-Rueda et al. 2016; Nakamura 1988; Teixeira et al. 2006). These include sibling competition (Ganeshaiah and Uma Shaanker 1988; Jerry and Carol 2015), poor pollination and fertilization (Miyajima et al. 2003; Shen et al. 2018; Xie et al. 2019), endosperm abortion (Sun et al. 2009; Pan et al. 2011), abnormal hormone metabolism (DeBruin et al. 2018; Daniela et al. 2020a, b; Okamoto and Omori 1991), and nutritional supply imbalance (Ji et al. 2019; Shen et al. 2020). *P. ludlowii* is self-compatible (Li et al. 1996; Tang et al. 2021), and the pollen fertility and germination rate of *P. ludlowii* are high (Jia et al. 2021). In this study, there was no abnormality in the process of pollination and fertilization, therefore the factor of poor pollination and fertilization could be excluded*.* During flowering and fruiting, the axillary buds on the flowering branches of *P. ludlowii* sprouted into the secondary branches, and the apical buds on the secondary branches differentiated (Yuan et al. 2021). Thus, the fruit development period coincided with the secondary branch growth period and the apical bud differentiation period. Consequently, the nutrients should be balanced and distributed among the three, and this may be one of the causes of ovule abortion. In addition, during the early and middle stages of embryo and endosperm abortion, the endosperm degenerated first, while the embryo remained intact, possibly because the embryo growth and differentiation mainly depend on the endosperm. As an important source of nutrients for embryo development, the endosperm can store nutrients, such as starch, lipids, and proteins (Gehring et al. 2004; Lopes and Larkins 1993; Stephen et al. 2008). When the endosperm degenerated, the embryo lacked nutrients and was aborted. In conclusion, the reasons for ovule abortion in *P. ludlowii* are complex and influenced by several factors. The findings of this study expand our understanding of the source and characteristics of ovule abortion, and the specific factors of ovule abortion could be an important future research direction focus.

Based on the renewal status of *P. ludlowii* forests, many naturally renewing seedlings were also found under suitable habitat conditions (Hong et al. 2017), indicating that this species does not have serious reproductive obstacles in the short term. The self-breeding rate of *P. ludlowii* is as high as 34.26–60% (Li et al. 1996; Tang et al. 2021), which provides some long-term reproductive protection and evolutionary potential for the species to adapt to a changing environment. However, whether inbreeding will cause genetic load that would lead to low competitiveness and abortion of some ovules remains to be investigated.

In recent years, with the development of local tourism and transportation, airports, new hotels, and roads have been built in Nyingchi, thereby causing disturbance and changes to the distribution areas of *P. ludlowii*. Presently, we are faced with the issue of harmonizing local economic development, arising from human activities in wild habitats, with the protection of endangered species, which should be urgently addressed.

## Data Availability

The authors declare that all data supporting the findings of this study are provided in full in the results section of this paper.
